# The first occurrence of *“Plesiochelyidae*” marine turtles in the Early Cretaceous of South America

**DOI:** 10.1186/s13358-025-00394-1

**Published:** 2025-08-25

**Authors:** Edwin-Alberto Cadena, Jorge D. Carrillo-Briceño, Dylan Bastiaans, Tandra Fairbanks-Freund, Loïc Costeur, Torsten M. Scheyer

**Affiliations:** 1 Escuela de Ciencias e Ingeniería, Grupo de Paleontología Neotropical Tradicional y Molecular (PaleoNeo), Universidad del Rosario, Cra 26 63C-69, 111211 Bogotá, Colombia; 2 Smithsonian Tropical Research Institute, Panama City, Panama; 3 Field Museum of Natural History, Chicago, USA; 4 Universität Zürich, Paläontologisches Institut, Zurich, Switzerland; 5 Naturhistorisches Museum Basel, Basel, Switzerland

## Abstract

Thalassochelydians represent one of the earliest radiations of coastal to marine-adapted turtles, spanning from the Jurassic to Cretaceous periods. This study describes *Craspedochelys renzi* sp. nov., a new thalassochelydian, “plesiochelyid” turtle from the Hauterivian stage of the Early Cretaceous in Colombia. It is the youngest and the only known record of “*Plesiochelyidae*’ outside Europe. The holotype, housed at the Naturhistorisches Museum Basel, was rediscovered after over 60 years and includes a partial shell, hindlimb bones, and caudal vertebrae. *C. renzi* shares key traits with “*Plesiochelyidae*”, including a V-shaped posterior plastral lobe lacking an anal notch; an indentation at the hypoplastra-xiphiplastra contact; an “intermediate” bone between neural 8 and suprapygal 1; a fully ossified carapace and bridge; and absence of carapacial fontanelles. Within “*Plesiochelyidae*”, it is assigned to *Craspedochelys* by its broader carapace, with an estimated length/width ratio of 4.12 for left costal 4, similar to other species in the genus; a relatively shorter plastron; and wider hyoplastra. Geological evidence links the specimen to the Moina Formation, a shallow marine deposit from the Hauterivian. This discovery expands the genus’ spatial and temporal range, underscoring its evolutionary and paleobiogeographic significance. It highlights the importance of museum collections in re-evaluating long-overlooked specimens, enriching our understanding of past biodiversity and turtle dispersal patterns.

**Supplementary Information:**

The online version contains supplementary material available at 10.1186/s13358-025-00394-1.

## Introduction

**Fig. 1:**
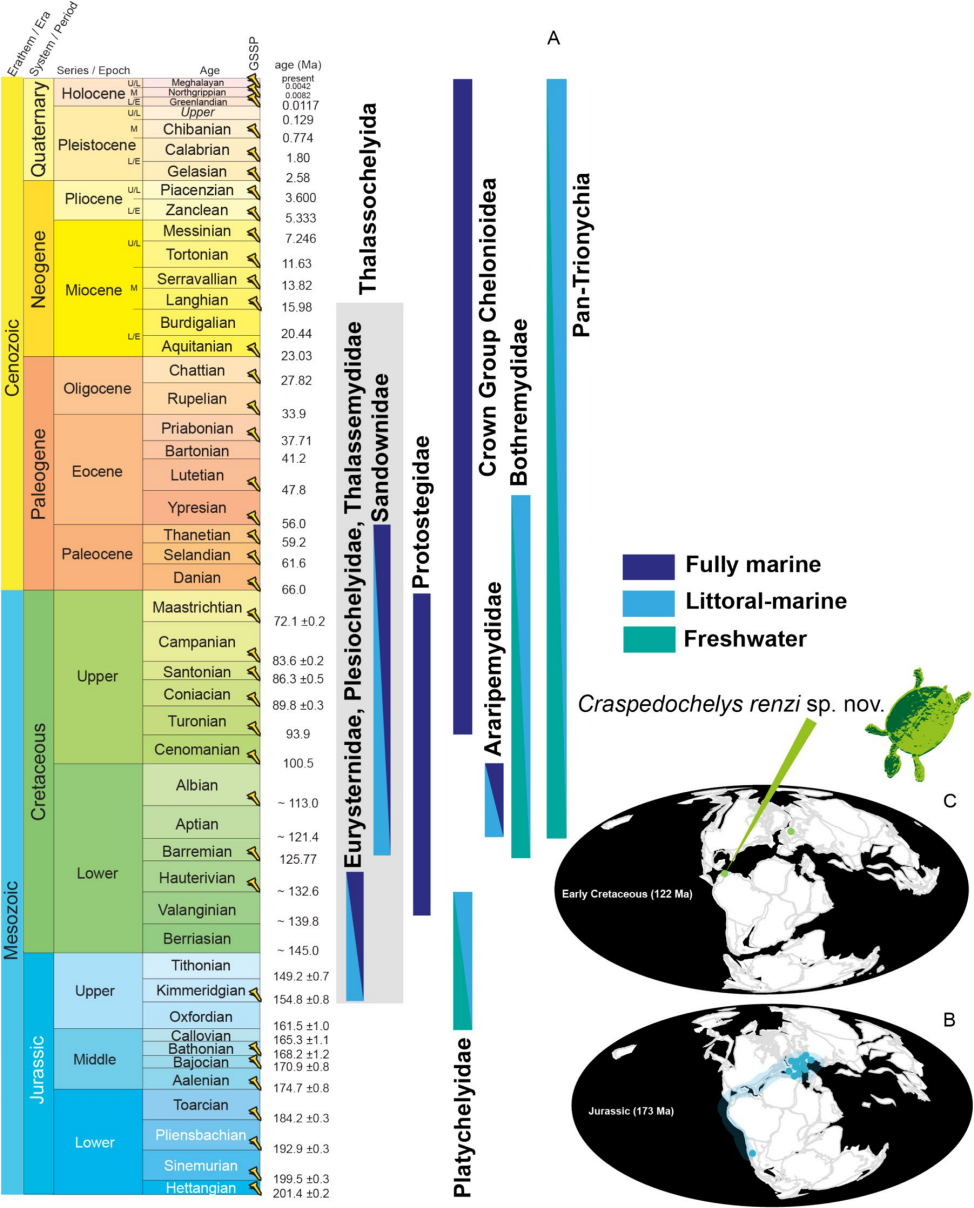
**A** Geologic time and ranges of different clades of turtles adapted to fully marine or littoral-marine conditions. Paleotectonic reconstruction during the Middle Jurassic (173 Ma) (**B**), showing the known occurrences of thalassochelydians (sensu Anquetin et al., 2017), and during the Hauterivian (122 Ma) (**C**). Paleotectonic reconstructions are based on data from the Paleobiology Database (paleobiodb.org)

Throughout their evolution, various unrelated groups of turtles have developed adaptations to live in marine and littoral environments (Evers et al., 2019; Joyce et al., 2021) (Fig. 1A). One of these groups is called *Thalassochelydia*, formed traditionally by three Jurassic families: Eurysternidae, Plesiochelyidae, and Thalassemydidae, with unresolved and controversial phylogenetic relationships (Anquetin et al., 2015, 2017). Recently, two other marine to littoral groups, Sandownidae (Cretaceous to Paleocene) and Protostegidae (Cretaceous), have also been hypothesized as potentially being part of *Thalassochelydia* (Evers & Joyce, 2020; Joyce et al., 2021). This hypothesis is still pending validation and phylogenetic stability.

The fossil record of traditional thalassochelydians (sensu Anquetin et al., 2017) is almost exclusively restricted to the Late Jurassic (Oxfordian to Tithonian) of Europe, with only one species from South America, *Neusticemys neuquina* from the Tithonian of Argentina (Anquetin et al., 2017; de la Fuente et al., 2021; Fernández & de la Fuente, 1988; Pérez-García et al., 2023) (Fig. 1B). An Early Cretaceous (Valanginian) record from Sainte-Croix, Canton of Vaud, Switzerland, attributed as ‘*Tropidemys (Chelonia) valanginiensis*’ (Pictet & Campiche, 1858–1860; Rütimeyer, 1873), has been considered dubious because of its uncertain stratigraphic provenance (locality and horizon), as well as its highly fragmentary nature, and can only be identified as *Tropidemys* sp. (Anquetin et al., 2017; Püntener et al., 2014). Other Early Cretaceous record of thalassochelydians include a nearly complete cranium from the Berriasian of England (Anquetin & André, 2020), and *Hylaeochelys belli* from the Berriasian to Valanginian of United Kingdom and Germany (Hirayama et al., 2000; Lydekker, 1889; Mantell, 1844; Milner, 2004; Pérez-Garcia & Ortega, 2014; Pérez-García et al., 2023, references therein). The taxonomic placement of *H. belli* remains controversial, as it is unclear whether it truly belongs to *Thalassochelydia* (see Anquetin & André, 2020), and it is sometimes excluded from the group (Anquetin et al., 2017; Evers & Benson 2018).

Of the three families that constitute the thalassochelydians, “Plesiochelyidae” is one of the most diverse, including at least ten species in four different genera (*Craspedochelys, Plesiochelys, Portlandemys,* and *Tropidemys*), according to Anquetin et al. (2017). Shells of “plesiochelyids” can be distinguished from other thalassochelydians by exhibiting large sizes (carapace length of 400–550 mm), the absence of carapacial fontanelles in adults, an osseous bridge, and, at most, a central plastral fontanelle (Anquetin et al., 2017). Here, we describe a new species of the genus *Craspedochelys* Rütimeyer, 1873, based on a remarkable fossil turtle shell and some of its postcranial bones from Colombia. This fossil represents the youngest record known so far for thalassochelydians (sensu Anquetin et al., 2017) worldwide, from the Hauterivian, and the second record of the group outside of Europe (Fig. 1C), accompanied by a particular story of how we rediscovered it after more than half a century. We also discuss the systematic paleontology, possible phylogenetic position, and paleobiogeographical implications of this new species.

## Materials and methods

### Recovery of the fossil specimen

**Fig. 2:**
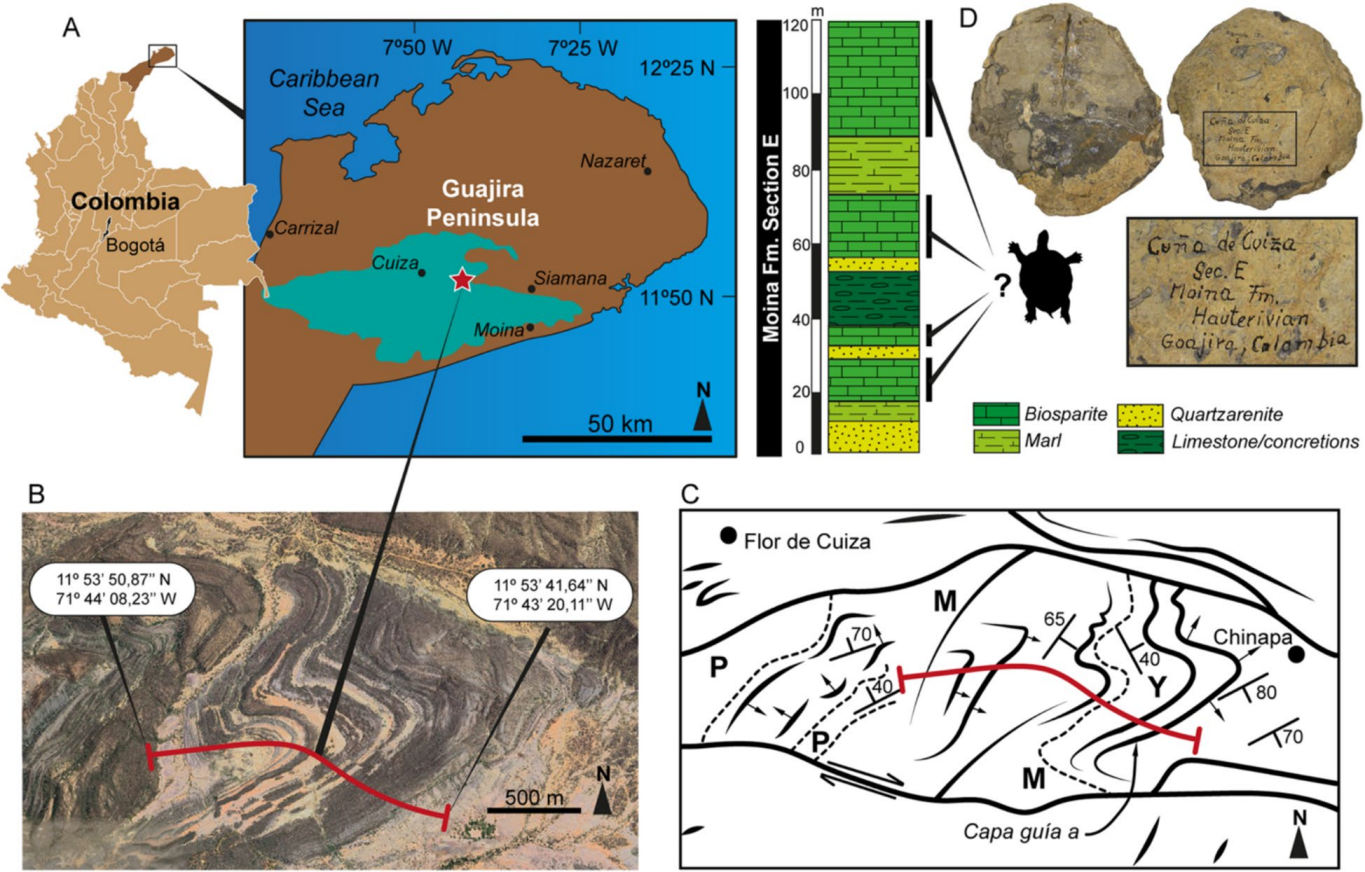
**A** Location of the Guajira Peninsula and the Cuña de Cuiza region (highlighted in green), including the exact locality based on a satellite image (Google Earth 2024) (**B**) and a geologic map (**C**) corresponding to this region. The geologic map shows the stratigraphic section where *Craspedochelys renzi* sp. nov. was collected by Otto Renz, redrawn from Renz (1960). (**D**) Stratigraphic section “E”, highlighted in red in (**B**, **C**), of the Moina Fm., indicating the possible horizons from which the new species described here have been collected. This section has been redrawn from Renz (1960) and includes a photograph of the specimen as rediscovered in the collections of the NMB, along with the original information on its collection. *Abbreviations in figure* **C:** M, Moina and P, Palanz formations

The fossil specimen NMB SA.M.19 was initially discovered and collected by Swiss geologist Otto Renz during a fieldwork expedition to the Cuña de Cuiza region, Guajira Department of Colombia (Fig. 2A–C), in the 1950s. The fossil, embedded in a biosparite limestone matrix, was appropriately labeled with information about the locality and formation (Fig. 2D). Subsequently, it was housed at the paleontological collections of the Naturhistorisches Museum Basel, Switzerland, where it remained forgotten for more than 60 years in the cabinets of fossil invertebrate collections. Recently, two of the co-authors (JDCB, LC), during an examination of fossil collection from Latin America in the NMB, found it and brought it to the attention of the co-authors, for an opinion about it, followed by its description here.

### Fossil preparation and study

The fossil specimen was prepared at the paleontological preparation lab of the Naturhistorisches Museum Basel in 2019 using airtools from Hardy Winkler and sandblasted with natrium bicarbonate. Paraloid B-72 in acetone was used as a consolidant and adhesive. The original preparation assignment was to remove the oyster from the carapace and to enhance details on the shell. While removing sediment with the air tool, the post-cranial bones were discovered. We photographed the fossil using a Nikon DS 5000 camera and close-ups of some regions of the fossils using a Keyence digital-microscope vhx-7000 with VHX-7100 fully integrated head at the Department of Paleontology of the University of Zurich.

### Phylogenetic analyses

We added NMB SA.M.19 specimen to the character-taxon matrix of Joyce et al. (2021), along with *Craspedochelys jaccardi*, the most complete species of the *Craspedochelys* genus, resulting in a matrix of 100 taxa in total. We made some changes on the characters (Supplementary file S1), including adding an extra character state to character 261, correcting the coding of characters 219 and 223 for some taxa, and adding a new character (character 357) to include the intermediate element (bone) described in Anquetin et al. (2014), which is of relevance for thalasochelydians. This brings the total to 357 characters (Supplementary file S2).

We conducted the phylogenetic analyses using the program TNT Version 1.6 (Goloboff & Morales, 2023). For the first analysis, we applied parameters like those in Joyce et al. (2021): memory increased to 10000 trees, collapsing rule 1 (minimun length = 0), *Progranochelys quenstedtii* as the outgroup, and a backbone constraint based on molecular phylogeny for extant taxa following Pereira et al. (2017) (Supplementary file S3), leaving other operation taxonomic units (OTUs) as floaters. Following Ezcurra (2024) and Oriozabala et al. (2025), we applied implied weights attached to a concavity value (*k*) of 11, which Ezcurra (2024) suggests is a suitable range for datasets with 100 terminal taxa. We performed a "new technology search" approach. This involved activating the ratchet, tree drifting, sectorial searches, and tree fusing algorithms. The search was configured with a driven search set at 7 initial additional sequences (set level = 30) and a best-hit score threshold of 30, followed by a second round of tree bisection and reconnection (TBR) on the most parsimonious trees (MPTs) in RAM to identify all MPTs. Finally, we obtained a strict consensus tree from of all the MPTs and calculated the Consistency Index (CI) and Retention Index (RI) using the script STATS.RUN. We also obtained Bremer support values using TNT. (Supplementary file S3). Since some polytomies were found in the strict consensus tree from the initial analysis, we searched for up to five 'wildcard' taxa using the pruned trees option under the trees/comparison’s menu in TNT. This analysis identified *Argillochelys cuneiceps* as wildcard taxa. This taxon was then inactivated, along with the entire clade formed by Pleurodira (*Chelus fimbriatus, Phrynops geoffroanus, Elseya dentata, Chelodina longicollis, Araripemys barretoi, Podocnemis unifilis, Pelomedusa subrufa*, and *Galianemys whitei*), as the matrix lacks a significant number of characters focused on this clade, making its phylogenetic position unstable in the initial analysis. As indicated in Joyce et al. (2021), NKMB Watt18/211 and additional material described therein was treated as a single OTU *Thalassemys bruntrutana*. We then repeated the analysis with the same settings as in the initial analysis, yielding a new consensus tree.

### Nomenclatural act

This published work and the nomenclatural acts it contains have been registered in ZooBank, the online registration system for the International Commission on Zoological Nomenclature (ICZN). The ZooBank LSIDs (Life Science Identifiers) can be accessed and the associated information viewed through any standard web browser by appending the LSID to the prefix http://zoobank.org/. The LSID for this publication is: urn:lsid:zoobank.org:act:9C50F995-6FE1-4366-B692-994AA9C83C64.

### Institutional abbreviations

NMB, Naturhistorisches Museum Basel, Basel, Switzerland.

#### Geologic and stratigraphic framework

Although we were unable to revisit the region where Otto Renz collected the specimen, we used the information written on the fossil (Fig. 2D). Using the geologic and stratigraphic report by Renz (1960), and satellite images from Google Earth Version 7.3.6.9345 accessed in December 2023, we have to relocated the stratigraphic sequence “E” of the Moina Formation (Fm.) (Renz, 1960), where the fossil was collected (Fig. 2B–D).

The “E” section of the Moina Fm. as described by Renz (1960) represents a shallow marine sequence of rocks characterized by thick strata of biosparite limestones interbedded with marls, limestones with concretions, and quartzarenites (Fig. 2D). According to Patarroyo (2021) this unit was deposited under high energy influxes conditions. NMB SA.M.19 was preserved in a hard biosparite limestone, including abundant remains of trigonid (*Pterotrigonia* sp.) and ostreids (*Ceratostreon* sp.) bivalves, indicating that it could have originated from one of the four strata exhibiting this lithology in the section “E” of the Moina Fm. (Fig. 2D). The predominance of the ostreid *Ceratostreon* sp., ammonoids of the *Olcostephanus* genus and the agglutinated foraminifera *Choffatella sogamosae* indicate a Hauterivian age for the Moina Fm., similarly to what occurs in the stratigraphic section at the top of the Rosa Blanca Fm. in the middle of Magdalena Valley of Colombia (Patarroyo, 2021; Renz, 1960).

#### Systematic paleontology

*Testudinata* Klein, 1760; sensu Joyce et al., 2020

*Thalassochelydia* Anquetin et al., 2017

“*Plesiochelyidae*” Baur, 1888; sensu Anquetin et al., 2017

*Craspedochelys* Rütimeyer, 1873

*Craspedochelys renzi* sp. nov.

(Figs. 3, 4, 6).

**Fig. 3:**
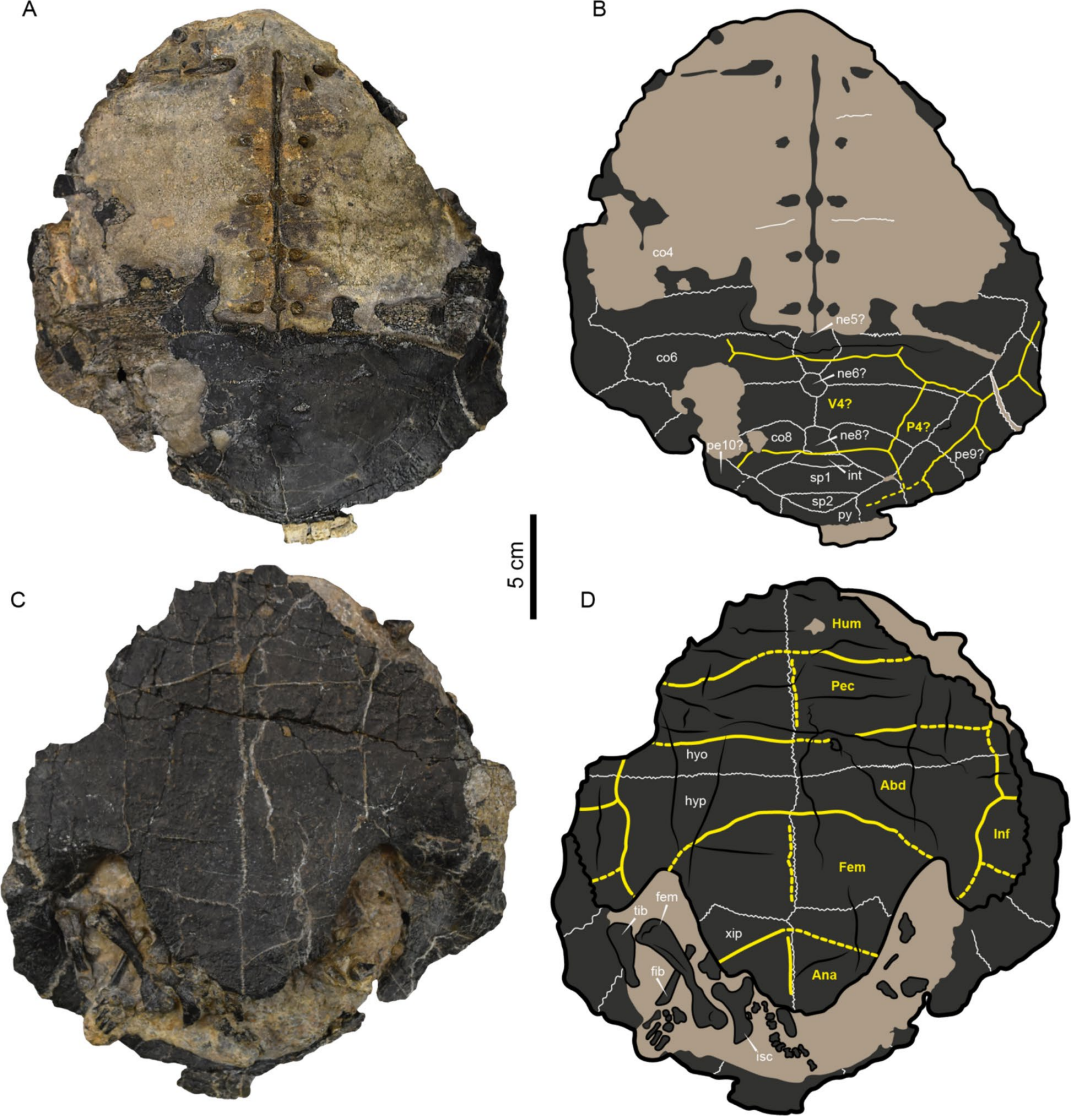
*Craspedochelys renzi* sp. nov. represented by an articulated shell with some postcranial bones. **A**, **B** Carapace in dorsal view and interpretative line drawing; **C**, **D** Plastron in ventral view, with some postcranial bones and their respective interpretative line drawing. Bones are indicated by dark grey, sutures by white lines, sulci by yellow lines, fractures and margins by black lines, and the rock matrix by light ochre shadows. *Abbreviations:* Abd, abdominal scute; Ana, anal scute; Co, costal bone; Fem, femoral scute; fem, femur; fib, fibula; Hum, humeral scute; hyo, hyoplastron; hyp, hypoplastron; Inf, inframarginal scute; Int, intermediate bone; isc, ischium; ne, neural bone; P, pleural scute; pe, peripheral bone; Pec, pectoral scute; py, pygal bone; sp, suprapygal bone; tib, tibia; V, vertebral scute; xip, xiphiplastron

**Fig. 4:**
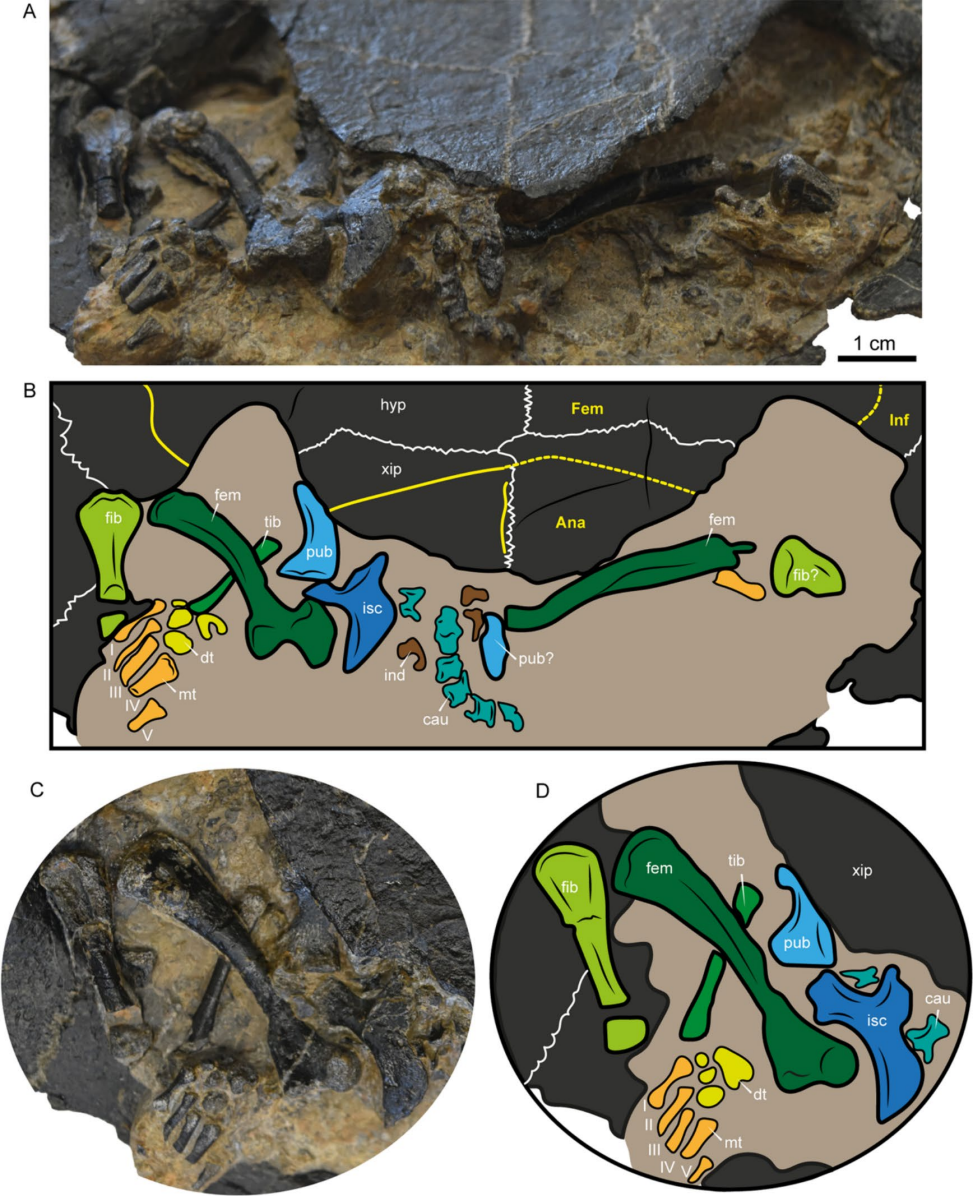
**A**,** B** *Craspedochelys renzi* sp. nov. posteroventral view of the posterior plastral lobe, and postcranial bones and their interpretative line drawing; **C**, **D** close-up of the right hindlimb bones, pes, and pelvic girdle elements preserved and their interpretative line drawing. Bones are indicated by dark grey, sutures by white lines, sulci by yellow lines, fractures and margins by black lines, and the rock matrix by light ochre shadows. *Abbreviations:* Ana, anal scute; cau, caudal vertebra; dt, distal tarsal; Fem, femoral scute; fem, femur; fib, fibula; hyp, hypoplastron; ind, indeterminate bone; Inf, inframarginal scute; isc, ischium; mt, metatarsal; pub, pubis; tib, tibia; xip, xiphiplastron

*Etymology*. In honor of the Swiss geologist Otto Renz, who discovered and collected the fossil turtle Otto Rentz's legacy also includes important geological and paleontological studies of the Cretaceous of Colombia and Venezuela (Renz, 1982, and references therein).

*Holotype*. NMB SA.M.19 partially preserved articulated shell (bone component of the posterior carapace, infilled of the mid-anterior carapacial cavity, and nearly complete plastron); most of its right hindlimb bones including the femur, the tibia, the fibula, autopodium bones; some bones of the left hindlimb including the femur and a fibula? portion; both pubes and right ischium; and at least six caudal vertebrae.

*Type locality and horizon*. “E” section (between 11º53′50.87’’N, 71º44′08.23’’W and 11º53′41.64’’N, 71º43′20.11’’W) (Fig. 1B, C) Moina Fm. following Renz (1960), Cuña de Cuiza, Guajira Department, Colombia (Fig. 2A). Indeterminate stratigraphic horizon (Fig. 2D).

*Diagnosis*. *Craspedochelys renzi* shares the following combination of characteristics with other thalassochelyians, particularly with members of “*Plesiochelyidae*”: (1) a V-shaped posterior plastral lobe, reduced in length and without anal notch; (2) an indentation of the sutural contact between the hypoplastra and xiphiplastra; (3) occurrence of an "intermediate" bone between the last neural (neural 8) and suprapygal 1; 4) completely ossified carapace and bridge to the plastron, and (5) lack of carapacial fontanelles. Inside “*Plesiochelyidae*”, it is attributable to *Craspedochelys* and excluded from *Plesiochelys* by: (1) a broader carapace, which appears to have been approximately as wide as it was long, as indicated by an estimated (as preserved) length/width ratio of 4.12 (103/25 mm) for the left costal 4—similar to other species of the genus with values greater than 4 (Anquetin et al., 2014, Table 3); (2) a relatively shorter plastron, and (3) proportionally wider hyoplastra. It is excluded from being part of *Tropidemys* by (1) its wider vertebral scutes, and (2) absence of neural keels. It differs from all other “plesiochelyids” by (1) suprapygal 2 small and in a nearly triangle shape, instead of being trapezoidal or pentagonal, and in posterior contact only with the pygal, without reaching the posterior-most peripheral bones, and (2) from all other the specimens of *Craspedochelys* spp., by exhibiting an incomplete neural series, where costals 7 meet medially, but costals 8 are separated by an isolated neural, potentially neural 8.

*Description*.—The shell of *Craspedochelys renzi* sp. nov.is three-dimensionally preserved thanks to the rock matrix that created a cast of its internal cavity (Fig. 3). As preserved, the shell measures 25.5 cm in length and 23.1 cm in width. The cortical bone of both carapace and plastron exhibits a smooth to slightly eroded surface in some regions.

*Carapace.* The original bony component of the carapace is preserved only at the most posterior part, with some missing portions of the left peripherals. A rock matrix cast of the mid-anterior portions of the carapace preserves, in anatomical position, parts of the axillary buttresses and the medial rib projections of the costals, indicating that there were eight pairs of costals. The most ventral portions of the thoracic ribs are also preserved along the midline of the carapacial cast (Fig. 3A, B). Although the complete shape of the carapace is not possible to establish, as preserved, it is evident that it was nearly as wide as long. The neural series is interrupted by a medial contact of costals 7. A neural bone is located right before the intermediate bone, possibly neural 8?. Two neurals, possibly 5 and 6? are nearly hexagonal and restricted between costals 5 to 7, suggesting that neural 7 is absent in the specimen or at least not expressed dorsally. The intermediate bone is nearly trapezoidal in shape and restricted between costals 8, suprapygal 1 and neural 8?. There are two suprapygal bones, being suprapygal 1 the larger and exhibiting an elongated hexagonal shape. The suprapygal bone 2 is triangular and restricted between the suprapygal 1 and the pygal. Most of the posterior edge of the pygal bone is missing. The preserved costal bones (5 to 8) are rectangular elongated, slightly longer at their lateral edges. This seems to be the case also for the fully eroded costals 2 to 4, as some portions of the sutural contacts between them are preserved as scars in the rocky cast, in the particular case of the left costal 4, it has an estimated preserved length of 103 mm and a width of 25 mm. The posterior peripherals 7 to 11 of both sides are preserved, squared in shape and most of them with their lateral edges broken, especially those from the left side. The sulci left by vertebral scutes 4 and 5 indicate that both were hexagonal in shape, much wider than long. Pleural scute 4 was trapezoidal in shape and much narrower than pleural scute 3. Marginal scutes were restricted to the peripherals, without reaching the costals.

*Plastron*. The plastron of *C. renzi* sp. nov.is nearly complete, missing only the most anterior portions of the anterior lobe, including the entoplastron and epiplastra. It measures 19.2 cm in length and 19.8 cm in width as preserved (Fig. 3C, D). The posterior plastral lobe exhibits a V-shape, lacking a medial anal notch. Each xiphiplastron has a nearly triangular shape, with an indentation at the sutural contact with the hypoplastron, near the lateral region of the bone. The hypoplastra are highly sutured to the carapace laterally and meet each other medially without the presence of fontanelles. The hyoplastra are the largest bones of the plastron. Additionally, the hyoplastra are flat, meet medially, and are strongly sutured to the peripherals laterally. The anterior-most margins of the hyoplastra are broken and indicate that the missing entoplastron was a small bone located very anteriorly in the anterior plastral lobe. The sulci left by the plastral scutes are marked on the bones and indicate that the anals were restricted to the xiphiplastral, the femorals were the longest scutes, the abdominals the shortest medially. There is an indication of at least three lateral inframarginal scutes in both sides of the plastron.

*Postcranial.* Several postcranial bones are preserved in *C. renzi* sp. nov. (Fig. 4). They are only mentioned here as a more detailed description based on CT scan analysis that will be presented in a separate publication currently in progress. The postcranial bones exposed after preparation of the specimen include both femora, portions of both fibulae, the right tibia, and the right ischium and pubis. Most of the elements of the right pes are also preserved, including three distal tarsals and five metatarsals. Additionally, at least six caudal vertebrae are preserved, nearly articulated.

## Results and discussion

### Phylogenetic results

**Fig. 5:**
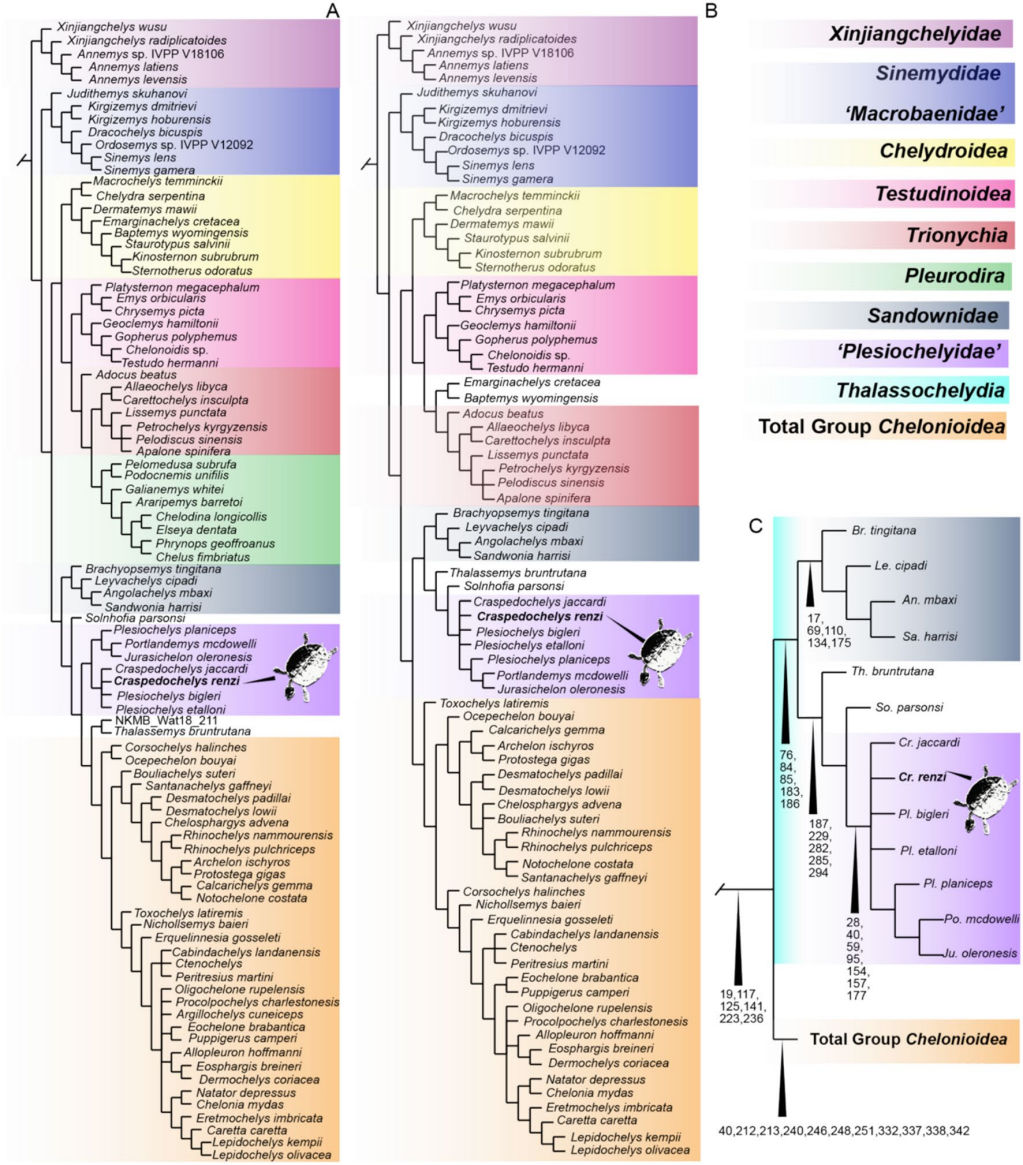
Phylogenetic hypotheses for the relationships of thalassochelydians, including *Craspedochelys renzi* (turtle icon) and other turtles. **A** Strict consensus of the first analysis (all taxa, molecular backbone forced); see Supplementary File S3 for the full tree, including consistency, and retention index values; **B** Strict consensus of the second analysis discussed in the text (excluding ‘wildcard’ taxa and Pleurodira, molecular backbone forced); see Supplementary File S3 for the full tree, including Bremer, consistency, and retention index values. The naming and color conventions of previously defined clades follow Joyce et al. (2021) are indicated in the upper left corner; **C** A map of the common synapomorphies from the second analysis (**B**) for the *Thalassochelydia* clade and the total group *Chelonioidea*, for comparison between two major clades of marine turtles. See Supplementary File S3 for the full map of synapomorphies

The strict consensus of the first analysis (all taxa) (Fig. 5A, Supplemental file S3) after a total of 227,694,186 rearrangements examined was obtained from 1134 trees most parsimonious trees (MPTs), with the following statistics: best score = 74.43010; tree length (TL) = 1789; consistency index (CI) = 0.239; and retention index (RI) = 0.672. The tree topology differs for some clades in contrast the one presented in Joyce et al. (2021). In particular, a monophyletic clade *Thalassochelydia* is not recovered in this hypothesis. Instead, it is split into three different clades: *Sandownidae*, ‘*Plesiochelyidae*’, and a third clade comprising *Thalassemys bruntrutana* and NKMB Watt18/211 (treated as a separate OUT in the first analysis), with *Solhnofia parsonsi* occupying an isolated position between them. Additionally, the placement of the aforementioned clades (*Sandwonidae*, ‘*Plesiochelyidae’*, and *Th. Bruntrutana*), differs from the topologies found by Evers & Benson (2018) and Joyce et al. (2021), instead indicating a closer relationship to the total group *Chelonioidea*. This affinity is also recovered in the second analysis (Fig. 5A, Supplemental File S3), supported by the following characters (non-exclusive synapomorphies) (Fig. 5C): ch 17(0), foramen stapedio-temporalis absent or weak, foramen stapedio- temporale concealed in dorsal view; ch 117(0), absence of a median contact of exoccipitals in the floor of the foramen magnum, excluding the basioccipital from the latter; ch 125(0), short ventral process of the prootic and without extensive posterior contact with the pterygoid; ch 141(2), parabasisphenoid, dorsum sellae high, i.e. a transverse ridge or wall of bone between the clinoid processes is present that projects dorsally at a high angle from the posteriorly positioned cup, separating the cup very clearly from of the anteriorly positioned rostrum basisphenoidale and sella turcica; ch 223(1) suprapygal 1 larger than suprapygal 2; and ch 236(1), presence of central plastral fontanelle. However, it is important to note that within each clade, these characters show variation or polymorphism. Another major difference and a problematic topological result given the molecular backbone enforced in the first analysis is the placement of the *Pleurodira* clade, which appears closer to *Trionychia*. This could be attributed to the limited inclusion of characters exclusive to *Pleurodira* in the character-taxon matrix, which was primarily focused on clades with marine adaptations.

In the second analysis, a monophyletic *Thalassochelydia* clade is recovered (Fig. 5B), including the same members as in the phylogenetic hypothesis of Joyce et al., (2021, Fig. 4), with the exception of the exclusion of *Ordosemys* sp. IVPP V12092. The *Thalassochelydia* clade is supported by the following characters (Fig. 5C): ch 76(0), anterior margin of the cavum tympanum formed entirely by the quadrate; ch 84(1), infolding ridge on the posterior surface of the quadrate ventral to the incisura columella auris present; ch 85(1), direction of cranial articular process of the quadrate with strong posterior inclination; ch 183(1), notch on posterior margin of coronoid present; ch 186(1), coronoid process relatively high, process is dorsally or posterodorsally pointed. The “*Plesiochelyidae*” clade is supported by the following characters: ch 27(0), medial process of jugal ventral to orbit weakly developed or absent, jugal contacts only the maxilla; ch 40(1), squamosal-quadrate contact wide open; ch 59(1), maxilla triturating surface with labial and lingual ridge; ch 95(1), pterygoid, foramen palatinum posterius present, but open laterally; ch 154(0), formation of the foramen posterius canalis carotici interni, parabasisphenoid involvement present; ch 157(1), hyomandibular branch of the facial nerve contained in a sulcus or separate canal paralleling the canalis cavernosus; and ch 177(0), dentary, lingual (tomial) ridge prominent. In both analyses, *Craspedochelys renzi* sp. nov. is found forming a polytomy within “*Plesiochelyidae*”, alongside *C. jaccardi*, *Plesiochelys bigleri*, and *Pl. etalloni*. This polytomy could potentially be resolved through the inclusion of new characters—for example, the length-to-width ratio of costal 4, which has proven useful in distinguishing *Craspedochelys* spp. From *Plesiochelys* spp. (see Anquetin et al., 2014, Table 2). However, this requires direct examination of the relevant specimens and, for that reason, has not been incorporated at this stage.

### Evolutionary and paleobiogeographical implications

The discovery and description of *Craspedochelys renzi* sp. nov. highlight the evolutionary persistence of diagnostic characteristics of thalassochelydians, particularly of “*Plesiochelyidae*”, —most notably the presence of an "intermediate" bone between neural 8 and suprapygal 1. A characteristic extensively documented previously in specimens from the Jurassic of Switzerland (Anquetin et al., 2014), and now documented in *C. renzi* sp. nov. from the Hauterivian. The incomplete neural series in *C. renzi* sp. nov., where costals 7 meet medially but costals 8 are separated by an isolated neural, represents an intriguing anatomical variation within the genus. This contrasts, for example, with the condition observed in the holotype specimen of *C. jaccardi* (Anquetin et al., 2014; Fig. 4), in which both costals 7 and 8 meet medially and a reduced neural 7 is present. Future discoveries of additional specimens of *C. renzi* sp. nov. will help determine whether this condition represents a diagnostic feature of the species or an individual anatomical anomaly.

**Fig. 6:**
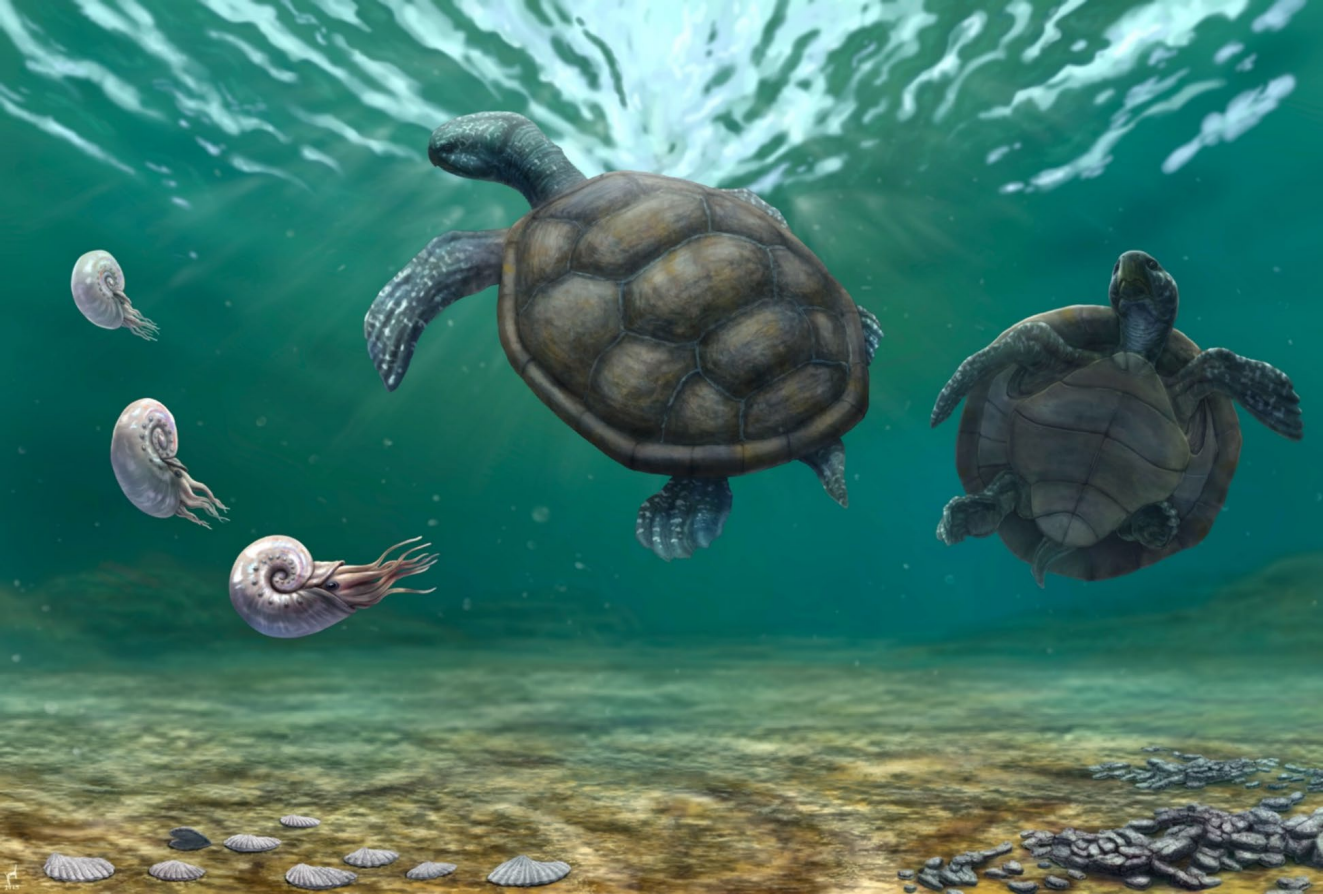
Artistic reconstruction of *Craspedochelys renzi* sp. nov. and the shallow marine ecosystem where inhabited. The forelimbs with a flippler like shape is based on the most complete thalassochelydian known so far, *Thalassemys bruntrutana* (Joyce et al., 2021). Illustration by Juan Giraldo

*Craspedochelys renzi* sp. nov. from the Hauterivian of Colombia represents a significant expansion of the geographic and temporal range of “*Plesiochelyidae*”, marking the first record for this clade outside of Europe (Fig. 1B, C). Furthermore, it provides evidence that “plesiochelyids” persisted in shallow marine environments, such as those interpreted for the Moina Fm., throughout much of the Early Cretaceous (Fig. 1A). This discovery also raises the possibility of temporal and ecological overlap between “plesiochelyids” and the earliest protostegids, as suggested by Valanginian records of the latter from the Rosa Blanca Formation in Colombia (Cadena & Combita-Romero, 2024). The Rosa Blanca Formation, which spans the Valanginian to Hauterivian, is considered stratigraphically equivalent to the Moina Formation within the same basin during the Lower Cretaceous (Patarroyo, 2021). Thus, The co-occurrence of trigonid and ostreid bivalves, along with ammonites, suggests a dynamic and nutrient-rich environment that may have provided suitable ecological conditions for a turtle adapted to coastal and shallow marine settings. These conditions are represented in an artistic reconstruction in Fig. 6, which is based on the well-preserved *Thalassemys bruntrutana* specimen NKMB Watt18/211 (Joyce et al., 2021)—one of the most complete thalassochelydians known to date—with modifications to reflect the shell and plastron morphology observed in *C. renzi* sp. nov.

## Conclusions

The discovery of *Craspedochelys renzi* sp. nov. represents a significant contribution to the understanding of thalassochelydian, particularly of the “plesiochelyid” turtles, extending their geographic range to northern Gondwana and their temporal range into the Hauterivian. This finding underscores the importance of reevaluating historical collections and highlights the potential for future discoveries in underexplored regions like northern South America. The presence of *C. renzi* sp. nov. in the Moina Fm. further emphasizes the complex paleobiogeographic history of costal and marine turtles during the Early Cretaceous. It also offers new insights into the evolutionary dynamics of *Thalassochelydia* and the still-controversial and unstable phylogenetic relationships within the group, issues that warrant further, in-depth investigation in future studies.

## Supplementary Information

Additional file 1.

Additional file 2.

Additional file 3.

## Data Availability

No datasets were generated or analysed during the current study.
