# Supplementary material for: The first occurrence of “Plesiochelyidae” marine turtles in the Early Cretaceous of South America
Source: Swiss J Palaeontol. 2025 Aug 25;144(1):52. doi: 10.1186/s13358-025-00394-1 (PMC12375516; doi:10.1186/s13358-025-00394-1)
Supplement: Supplementary file 1 — Additional file 1. [file 13358_2025_394_MOESM1_ESM.docx]

**New characters and modifications**

***State character modification***

**Character 261:** Xiphiplastra, articulation with hypoplastron: 0 = the xiphiplastra articulate with the hypoplastra along an anteriorly facing margin, forming a mediolaterally broad suture; 1 = the xiphiplastra have an elongate anterolateral process articulating along the posterolateral margin of the hypoplastron, resulting in an oblique suture, and the hypoplastra extend posteriorly along the anteromedial margin of the xiphiplastra: 2= on the ventral surface the xiphiplastral have a short elongate process or indentation located laterally approximately at the three quarters of the sutural line, creating a most lateral oblique sutural line.

*Remarks:* State 2 added to include the condition exhibited by most of the talassochelydian turtles.

***Corrected coding on the Joyce et al., 2021 Thalasochelydians matrix***

**Character 219.** Costals, costal 9: 0 = present; 1 = absent.

Coded as “0” for *Plesiochelys etalloni* and *Pl. bigleri.*

Corrected to “1”, based on the specimens figured in Anquetin et al. (2014, 2017). All of them lack of costal 9.

**Character 223.** Suprapygals, size between suprapygal 1 and 2: 0 = suprapygal 1 smaller than suprapygal 2; 1 = suprapygal 1 larger.

Coded as “0” for *Plesiochelys etalloni* and *Pl. bigleri.*

Corrected to “0&1”, for *Plesiochelys etalloni* based on the specimens figured in Anquetin et al., 2014

Corrected to “1”, for *Plesiochelys bigleri* based on the specimen figured in Rasselli & Anquetin, (2019).

***New characters***

**Character 357.** Intermediate element (bone): 0 = absent; 1 = present.

*Remarks :* following Anquentin et al. (2014), the intermediate element is an extra bone between the last neural and the suprapygal 1. It is present in *Craspedochelys,*

*Tropidemys, Thalassemys, Plesiochelys biglery,* and less common in some specimens of *Plesiochelys etalloni*
