# Supplementary material for: The first occurrence of “Plesiochelyidae” marine turtles in the Early Cretaceous of South America
Source: Swiss J Palaeontol. 2025 Aug 25;144(1):52. doi: 10.1186/s13358-025-00394-1 (PMC12375516; doi:10.1186/s13358-025-00394-1)

**Supplementary File S3. Detailed trees from the phylogenetic analysis**

**Figure S3A.** *Strict consensus tree from the first analysis*

Settings and results: Matrix (357x100, 16 states); Space for 10000 trees in memory; Max. Mem =3GB; Collapsing branches if supported ambiguously ("rule 1"); Implied Weighting is ON; Weighting strength is 11.00000; Constraints is ON, 28 positive tree-constraints, other taxa left as floaters; Random seed is 1. Search using “new technology search”. This included activating the ratchet, tree drifting, sectorial searches, and tree fusing algorithms, driven search set at 7 initial additional sequences (set level = 30), best score hit =30 times. We also performed a second round of TBR on the MPTs in RAM to search for all the MPTs. Total rearrangements examined: 1,516,725,031; Best score = 74.17123, 3 trees found after second round of TBR. Consistency index = 0.241; Retention index = 0.675. Tree length = 1714.


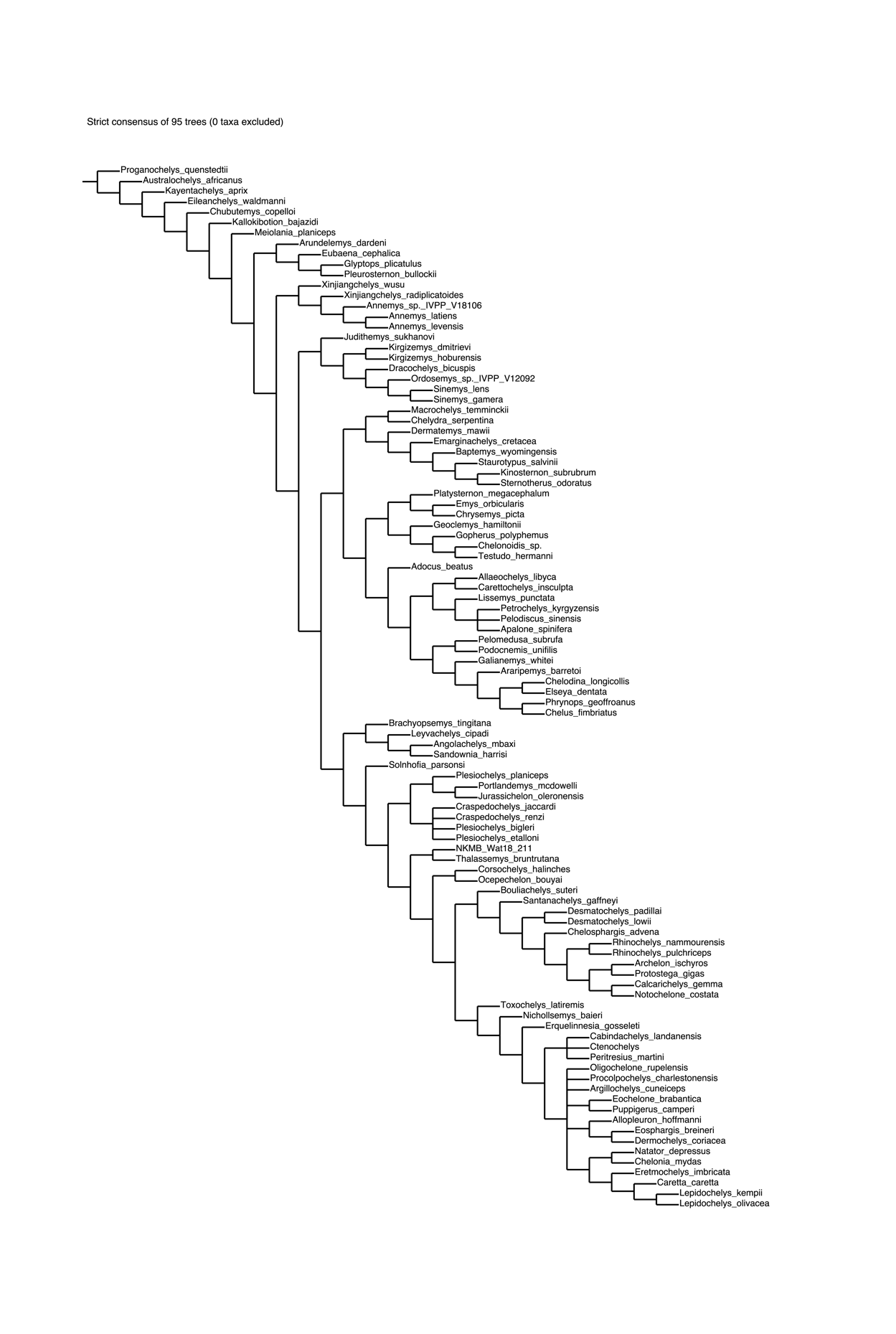


**Figure S3B.** *Strict consensus tree from the second analysis*

Settings and results: Equal settings as in the first analysis but removing (inactivating) “wildcard” taxa and the whole clade Pleurodida: *Argillochelys cuneiceps,* Pleurodira (*Chelus fimbriatus, Phrynops geoffroanus, Elseya dentata, Chelodina longicollis, Araripemys barretoi, Podocnemis unifilis, Pelomedusa subrufa*, and *Galianemys whitei*). Total rearrangements examined: 1,519,164. Best score (TBR): 64.09238. 9 trees found after second round of TBR. Consistency index = 0.277; Retention index = 0.731; Tree length = 1492.


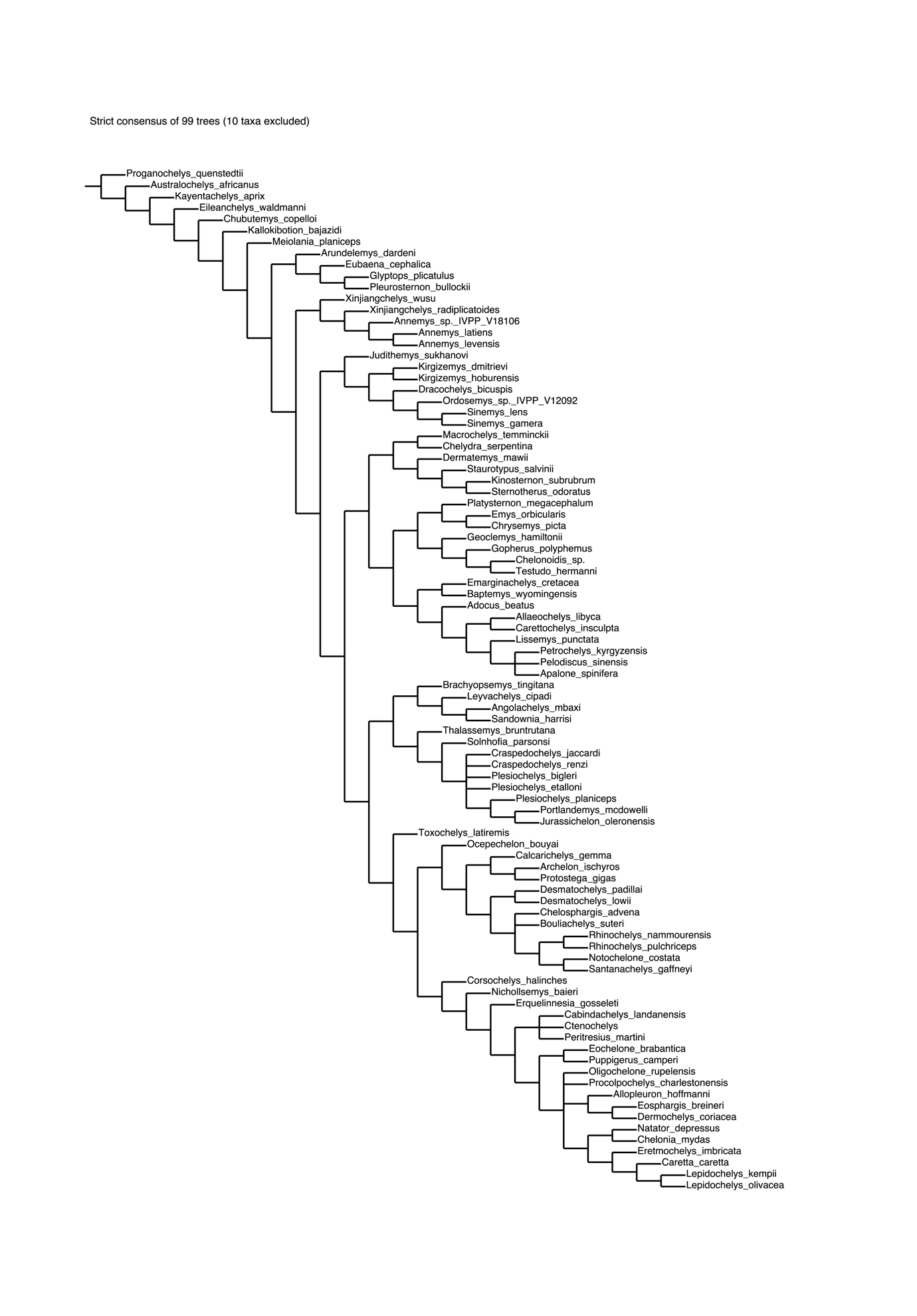


**Figure S3C.** *Strict consensus with combined Bremer via TBR of the second analysis*


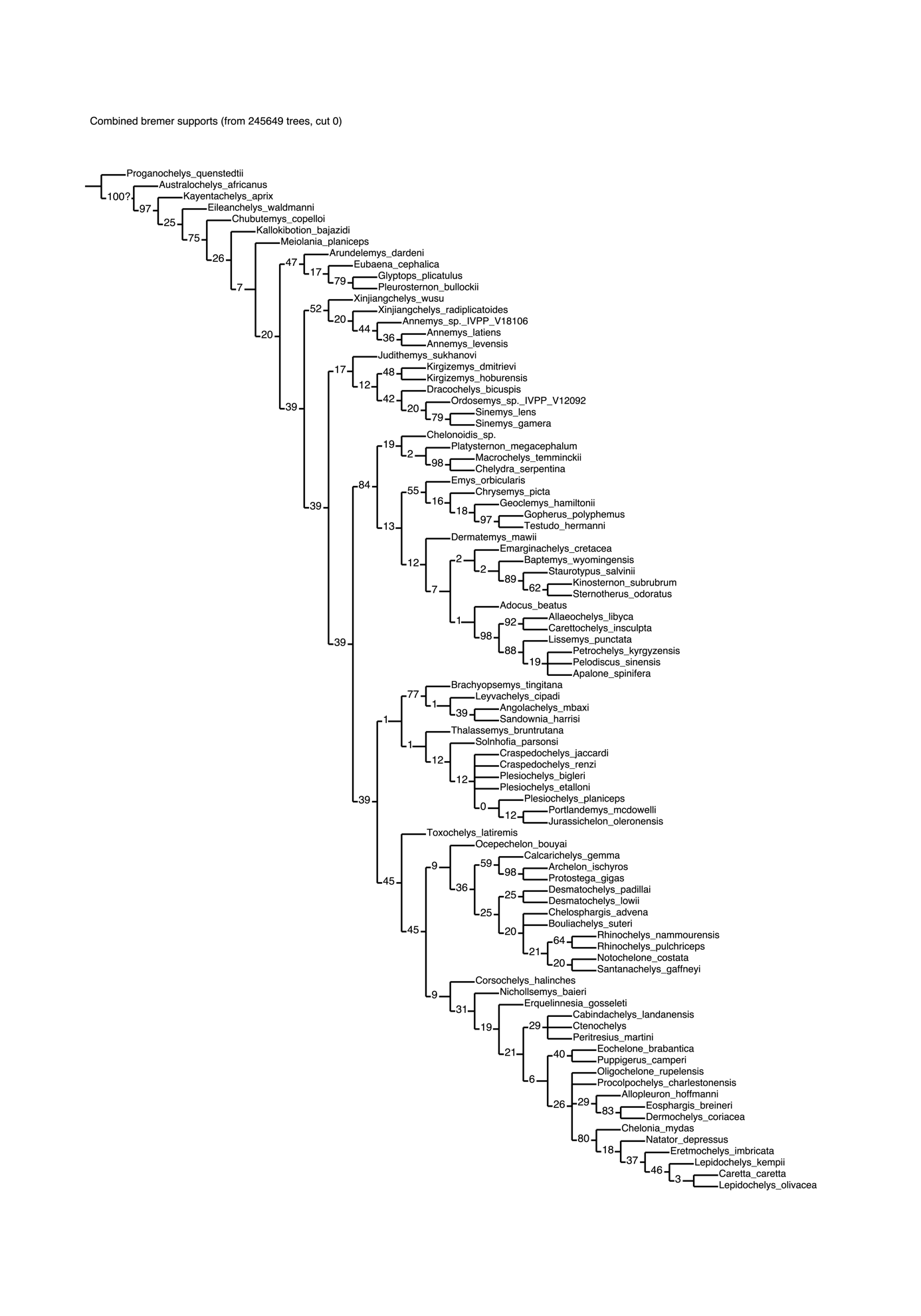


**Figure S3D.** *Full map of common synapomorphies resulted from the second analysis.*


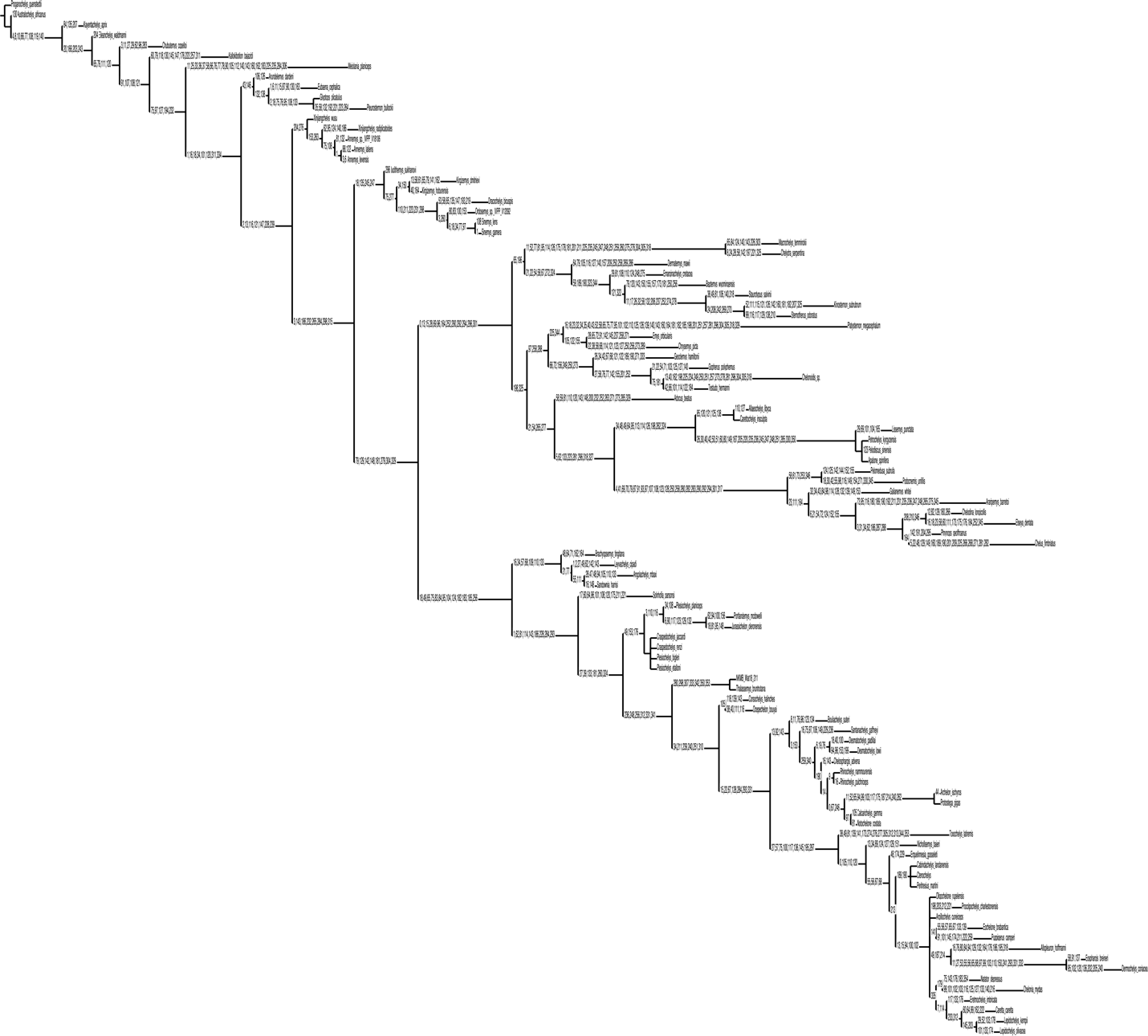

Supplement: Supplementary file 2 — Additional file 2. [file 13358_2025_394_MOESM2_ESM.docx]
